# Proteomic dataset of *Paracentrotus lividus* gonads of different sexes and at different maturation stages

**DOI:** 10.1016/j.dib.2016.06.037

**Published:** 2016-06-29

**Authors:** Stefania Ghisaura, Barbara Loi, Grazia Biosa, Maura Baroli, Daniela Pagnozzi, Tonina Roggio, Sergio Uzzau, Roberto Anedda, Maria Filippa Addis

**Affiliations:** a Porto Conte Ricerche S. r. l. – S.P. 55 Porto Conte – Capo Caccia, Km 8.400, Loc. Tramariglio, Alghero (SS), Italy; b IMC - International Marine Centre, località Sa Mardini, 09170 Torregrande (OR), Italy; c Dipartimento di Scienze Ecologiche e Biologiche (DEB), Università della Tuscia, Largo dell’Università snc, 01100 Viterbo (VT), Italy

## Abstract

We report the proteomic dataset of gonads from wild *Paracentrotus lividus* related to the research article entitled “Proteomic changes occurring along gonad maturation in the edible sea urchin *Paracentrotus lividus*” [1]. Gonads of three individuals per sex in the recovery, pre-mature, mature, and spent stages were analyzed using a shotgun proteomics approach based on filter-aided sample preparation followed by tandem mass spectrometry, protein identification carried out using Sequest-HT as the search engine within the Proteome Discoverer informatics platform, and label-free differential analysis. The dataset has been deposited in the ProteomeXchange Consortium via the PRIDE partner repository with the dataset identifier PRIDE: PXD004200.

**Specifications Table**

**Table:** 

Subject area	*Biology*
More specific subject area	*Proteomics*
Type of data	Tables
How data was acquired	Q-TOF hybrid mass spectrometer equipped with a nano lock Z161 spray source and coupled on-line with a NanoAcquity chromatography system (Waters)
Data format	Raw, processed
Experimental factors	*Proteome analysis of female and male sea urchin gonads during maturation stages* (*stage 1, 3, 4 and 6*) *(Paracentrotus lividus)*
Experimental features	(1) Protein extraction (mechanical disruption in TUC-based buffer) (2) Filter-aided sample preparation (FASP) (3) LC–MS/MS analysis
Data source location	Tramariglio, Alghero (SS), Italy; Torregrande (OR), Italy
Data accessibility	Data is within this article and available via the ProteomeXchange Consortium, dataset identifier PRIDE: PXD004200

## Value of the data

• The list of proteins identified in immature and mature gonads, together with their abundances, can be useful for understanding *P. lividus* biology as well as for finding markers of maturation and sex definition;
• The dataset might form the basis for the development of novel, rapid, and quantitative tools for an easier sexing and staging of *P. lividus*, both for monitoring the reproductive stages in the wild, as well as for monitoring productive cycles in aquaculture plants;
• The dataset presented here, together with protein abundance differences, might also be useful for comparing the proteins and processes of *P. lividus* gonads with those of other edible and non-edible sea urchins.

## 1 Data

Gonads from wild *P. lividus* of different sexes and at different maturation stages collected along coastal Sardinia were characterized with a shotgun proteomic approach. The protein identifications obtained in all samples are outlined in Table 1.

The list of all protein identifications obtained with the Proteome Discoverer software in female and male gonads at the four maturation stages is provided in Supplementary Table 1.

A differential analysis was carried out with a label-free approach by comparing all different groups according to maturation stages and sexes. Supplementary Table 1 reports the differential proteins (*P* value <0.05) observed in the four stages in female and male gonads passing the significance thresholds in at least one comparison (−0.5 >RNSAF >0.5). Other differential proteins not passing the thresholds are also included and indicated.

## 2 Experimental design, materials and methods

### 2.1 *Paracentrotus lividus* gonad samples

*P. lividus* individuals having diameter ≥50 mm (without spines) were gathered along coastal Sardinia and gonads were collected. Histological analysis [2], [3] was then carried out on one gonad to characterize sex and stage, while one gonad from each sea urchin per sex and stage was subjected to proteomic analysis [1].

### 2.2 Protein extraction and digestion

Gonad tissue was subjected to protein extraction and quantification as described in Ghisaura et al. [1]. Then, all protein extracts were processed with the filter-aided sample preparation (FASP) protocol [4], with some slight modifications [5]. Peptides were quantified with the BCA protein assay kit (Thermo Scientific - Rockford, IL).

### 2.3 LC–MS/MS and data analysis

Mass spectrometry analysis was carried out on a Q-TOF hybrid mass spectrometer (Waters) as described in Pagnozzi et al. [6].

Proteome Discoverer software (version 1.4.0.288; Thermo Scientific) was used to analyze the peak lists from the Q-TOF instrument after conversion into a MGF file. The workflow was as described in Ghisaura et al. [1]. Gene ontology and protein annotations were retrieved from UniProtKB (http://www.uniprot.org). All uncharacterized sequences were identified by homology through blasting on NCBI as non-redundant database (http://blast.ncbi.nlm.nih.gov/Blast.cgi).

Differential protein abundances of the different functional categories were estimated by the Normalized Spectral Abundance Factor (NSAF) [7]. The significance threshold RNSAF>0.5 or <−0.5 was applied. Student׳s *t* test (two-sample comparison, *p*<0.05) was used to evaluate the statistical significance of differences in protein abundance between logarithmized (normally distributed) NSAF values. The dataset was then deposited in the ProteomeXchange Consortium via the PRIDE partner repository [8], [9].

## Figures and Tables

**Table 1:** Summary of protein identifications obtained in all samples.

		**1F**^a^	**3 F**	**4 F**	**6 F**	**1 M**^b^	**3 M**	**4 M**	**6 M**
**Protein IDs**									
	**With 1 peptide**	202	301	202	135	221	391	262	142
	**With 2 peptides**	74	138	95	61	96	180	134	71
									
**Peptides**		462	724	514	398	546	959	727	464
**PSMs**		2121	2271	2251	1805	2324	3304	3200	2711

a F, females;

b M, males

## Footnotes

Appendix A Supplementary data associated with this article can be found in the online version at doi:10.1016/j.dib.2016.06.037.

## References

[1] Ghisaura S., Loi B., Biosa G., Baroli M., Pagnozzi D., Roggio T. (2016). Proteomic changes occurring along gonad maturation in the edible sea urchin *Paracentrotus lividus*. J. Proteom..

[2] Byrne M. (1990). Annual reproductive cycles of the commercial sea urchin *Paracentrotus lividus* from an exposed intertidal and a sheltered subtidal habitat on the west coast of Ireland. Mar. Biol..

[3] Siliani S., Melis R., Loi B., Guala I., Baroli M., Sanna R. (2016). Influence of seasonal and environmental patterns on the lipid content and fatty acid profiles in gonads of the edible sea urchin *Paracentrotus lividus* from Sardinia. Mar. Environ. Res..

[4] Wiśniewski J.R., Zougman A., Nagaraj N., Mann M. (2009). Universal sample preparation method for proteome analysis. Nat. Methods.

[5] Tanca A., Biosa G., Pagnozzi D., Addis M.F., Uzzau S. (2013). Comparison of detergent-based sample preparation workflows for LTQ-Orbitrap analysis of the Escherichia coli proteome. Proteomics.

[6] Pagnozzi D., Biosa G., Addis M.F., Mastrandrea S., Masala G., Uzzau S. (2014). An easy and efficient method for native and immunoreactive Echinococcus granulosus antigen 5 enrichment from hydatid cyst fluid. PLoS One.

[7] Zybailov B., Mosley A.L., Sardiu M.E., Coleman M.K., Florens L., Washburn M.P. (2006). Statistical analysis of membrane proteome expression changes in Saccharomyces cerevisiae. J. Proteome Res..

[8] Vizcaíno J.A., Deutsch E.W., Wang R., Csordas A., Reisinger F., Ríos D. (2014). ProteomeXchange provides globally coordinated proteomics data submission and dissemination. Nat. Biotechnol..

[9] Vizcaíno J.A., Csordas A., Del-Toro N., Dianes J.A., Griss J., Lavidas I. (2016). Update of the PRIDE database and its related tools. Nucleic Acids Res..

