# Supplementary material for: Proteomic dataset of Paracentrotus lividus gonads of different sexes and at different maturation stages
Source: Data Brief. 2016 Jun 29;8:824–7. doi: 10.1016/j.dib.2016.06.037 (PMC4960011; doi:10.1016/j.dib.2016.06.037)
Supplement: Supplementary file 1 — Supplementary material [file mmc1.docx]

**Conflict of interest**

The authors declare that they have no conflicts of interest.
